# Pocket-sized hand-held ultrasound for evaluating pediatric functional constipation by both novice and expert clinicians

**DOI:** 10.3389/fped.2025.1624070

**Published:** 2025-08-14

**Authors:** Pola Waissman, Ron Berant, Lisa Amir, Shimon Reif, Shmuel Springer

**Affiliations:** ^1^ Neuromuscular and Human Performance Laboratory, Department of Physiotherapy, Ariel University, Ariel, Israel; ^2^ Department of Pediatric Emergency Medicine, Schneider Childrens Medical Center of Israel, Petach Tikva, Israel; ^3^ Department of Pediatrics, Hadassah-Hebrew University Medical Center, Jerusalem, Israel

**Keywords:** pediatric functional constipation, pocket-sized ultrasound, point-of-care ultrasound, rectal wall thickness, transverse rectal diameter

## Abstract

**Background/Objectives:**

Pediatric functional constipation (PFC) is a global health concern. This study evaluates the utility of pocket-sized ultrasound (PsUS) as a tool for assessing PFC among clinicians with varying levels of ultrasound (US) experience. We assessed the validity of PsUS in measuring transverse rectal diameter (TRD) and rectal anterior wall thickness (RAWT) compared to conventional US and to evaluate agreement between expert and novice clinicians.

**Methods:**

In this cross-sectional study, TRD and RAWT were measured using conventional US and PsUS in 52 children (PFC: *n* = 28; non-constipated: *n* = 24), aged 4–14 years. Measurements were performed by an experienced and a novice clinician. Agreement and validity were assessed using intraclass correlation coefficients (ICCs), and diagnostic accuracy was evaluated using receiver operating characteristic (ROC) analysis.

**Results:**

High agreement was found between the experienced and novice clinicians in assessing PFC using conventional US (ICC for TRD = 0.98, 95% CI: 0.98–0.99; ICC for RAWT = 0.98, 95% CI: 0.97–0.99) and PsUS (ICC for TRD = 0.99, 95% CI: 0.97–0.99; ICC for RAWT = 0.97, 95% CI: 0.96–0.98). PsUS showed robust validity compared to conventional US (ICCs of 0.96 for TRD and 0.87 for RAWT). ROC analysis showed high diagnostic accuracy for PsUS at TRD [area under the curve (AUC) = 0.88, cut-off 30.25 mm] and RAWT (AUC = 0.91, cut-off 2.05 mm).

**Conclusions:**

These results suggest PsUS is a valid and reliable tool for assessing PFC, that can be used by clinicians with varying levels of US experience.

## 1 Introduction

Pediatric functional constipation (PFC) is a major global health problem with a reported median prevalence of 12% (1). PFC is a symptom-based condition typically characterized by infrequent bowel movements, hard and large stools, difficulty or pain during defecation, and fecal incontinence (2, 3). PFC is the most common diagnosis for constipated children (up to 95%) and is a diagnosis of exclusion without known organic causes (4–6). The major causative mechanism is considered to be withholding behavior leading to fecal retention (7). Stool retention in PFC is common and leads to rectal diameter expansion, which stretches the intestinal muscles and thickens their walls, reducing its effectiveness (8–10).

The North American and European Societies for Pediatric Gastroenterology, Hepatology, and Nutrition (NASPGHAN and ESPGHAN) (2) clinical guidelines recommend diagnosing PFC using the Rome IV Criteria. However, the self-reporting nature of the Rome IV Criteria presents difficulties, as it depends on obtaining accurate patient histories and bowel movement diaries (11, 12). Clinical guidelines also recommend digital rectal examination (DRE) for cases of unclear diagnosis, intractable constipation, or presence of alarm signs (13). Yet, its invasive nature can be stressful for the patient and make cooperation difficult (2). Additional tests such as colonic transit time and x-ray imaging have been suggested to enhance PFC diagnosis, but they face limitations such as radiation exposure and inconsistent results (14, 15). Although MRI is a radiation-free and non-invasive alternative that provides valuable anatomical and functional insights into the colon, its high cost, limited availability, long scan duration, and the need for patient cooperation make it unsuitable application in many routine pediatric conditions (16, 17). Beyond the challenges of diagnosing PFC, there is also a need for improved methods to monitor treatment progress. Therefore, alternative tools for PFC assessment should be explored.

Point-of-care ultrasonography (POCUS) offers a non-invasive, child-friendly approach to the assessment of PFC (18–22). By positioning the ultrasound (US) probe above the symphysis pubis, clinicians can measure transverse rectal diameter (TRD) and rectal anterior wall thickness (RAWT) (23, 24). These measurements often change with persistent constipation, likely due to chronic straining and increased intra-abdominal pressure (9, 18, 25–27). US studies report TRD values of 30.2–35.5 mm in constipated children vs. 19.8–23 mm in healthy controls (9, 24–27), with cutoff values ranging from 24.4 to 30 mm depending on age (9, 24, 26). RAWT in constipated children has been reported as approximately 27.7 mm (24). In addition, monitoring TRD and RAWT with POCUS may serve as a valuable indicator of the effectiveness of constipation treatment, as improvements in these outcome measures may correlate with more regular defecation (28). This form of follow-up may reassure and motivate patients (25), increase adherence to treatment and improve the long-term management of functional constipation (FC) (29). While US devices are widely utilized in a number of fields due to their well-established reliability and superior image quality (22), pocket-sized ultrasound (PsUS) devices have gained increasing acceptance in recent years, primarily owing to their portability, affordability, and ease of use (30–32). A systematic review has demonstrated that PsUS devices can deliver fast and accurate diagnoses in pediatric patients, with image quality comparable to that of conventional devices (33). However, the utility of PsUS in the assessment of PFC requires further investigation, particularly in terms of diagnostic accuracy compared with conventional devices and performance when used by clinicians with varying levels of US experience (34, 35).

The main objective of this study was to assess the level of agreement between expert and novice clinicians in the assessment of PFC using PsUS to investigate the feasibility of PsUS as a tool for assessment of constipation in children. In the first stage, we evaluated the validity of measuring TRD and RAWT with PsUS compared to measuring with conventional US which has already been proven effective for PFC assessment. Once the validity of PsUS was established, we assessed the agreement between experienced and novice clinicians (physiotherapist, PT) regarding these outcomes with both PsUS and conventional US. Finally, we established sonographic reference values and proof of temporal stability for these measurements to support the assessment of PFC.

## 2 Materials and methods

### 2.1 Study design and population

For this cross-sectional observational study, 52 children aged 4–14 years were recruited from the pediatric emergency department of XXX Children's Medical Center, XXX from November 2023 to February 2024. The sample was evenly divided between children with constipation symptoms who had been diagnosed with PFC by a pediatrician based on the Rome IV diagnostic criteria for FC and children admitted for other reasons. Children with malformations of the digestive system (e.g., Hirschsprung's disease, anorectal malformations), anatomical rectal anomalies, neurological diseases affecting bowel function (e.g., spina bifida, cerebral palsy), a history of gastrointestinal surgery, chronic systemic diseases, or metabolic disorders were excluded. Further exclusion criteria were severe psychiatric or behavioral conditions that could interfere with participation in the study, as well as open skin wounds in the symphysis pubis area that could interfere with US measurements. The hospital ethics committee approved the study (0135-23-RMC) and written parental consent was obtained prior to participation, with the option to withdraw participation at any time.

### 2.2 Data collection

**Figure 1:**
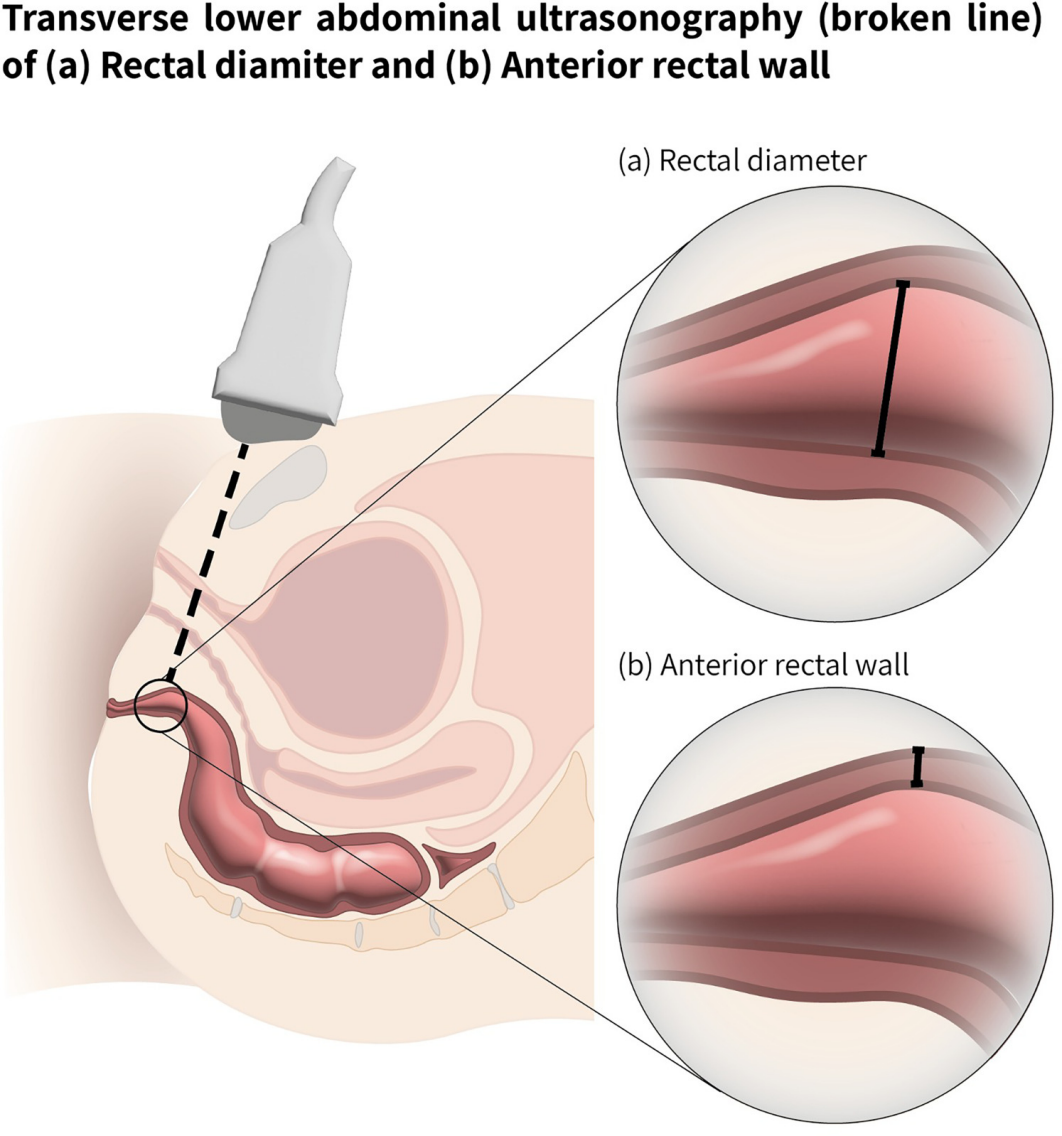
Ultrasound examination scheme for rectal measurements. Transverse lower abdominal ultrasonography, path shown with a broken line. **(a)** Transverse rectal diameter (TRD) shown with solid line **(b)** Rectal anterior wall thickness RAWT (shown with solid line).

All participants underwent four US examinations in random order to determine TRD and RAWT. The four examinations were performed sequentially using two different US devices - a conventional US system (Zonare Z.One Pro, Mindray, Mountain View, CA) and a pocket-sized hand-held US device (Philips Healthcare, Best, The Netherlands) both with an S4-1 MHz phased array probe. Each device was used by an experienced clinician (pediatrician with more than 10 years of US experience) and a clinician inexperienced with US (pediatric pelvic floor PT) who received a brief POCUS training program based on the “Four Elements of the Constipation POCUS Educational Program” developed by Matsumoto et al. (36) Each examiner performed the US assessments independently and was blinded to the other examiner's findings. Both devices used a convex US probe with a bandwidth of 2–5 MHz. TRD was measured in the transverse plane according to the guidelines of Klijn et al. (24), defined as the width between the right and left lateral aspects of the rectum. The RAWT was measured in the sagittal plane. The US probe was placed 2 cm above and parallel to the symphysis pubis, and tilted caudally by 10°–15° to accurately visualize and measure the rectal area (Figure 1).

**Figure 2:**
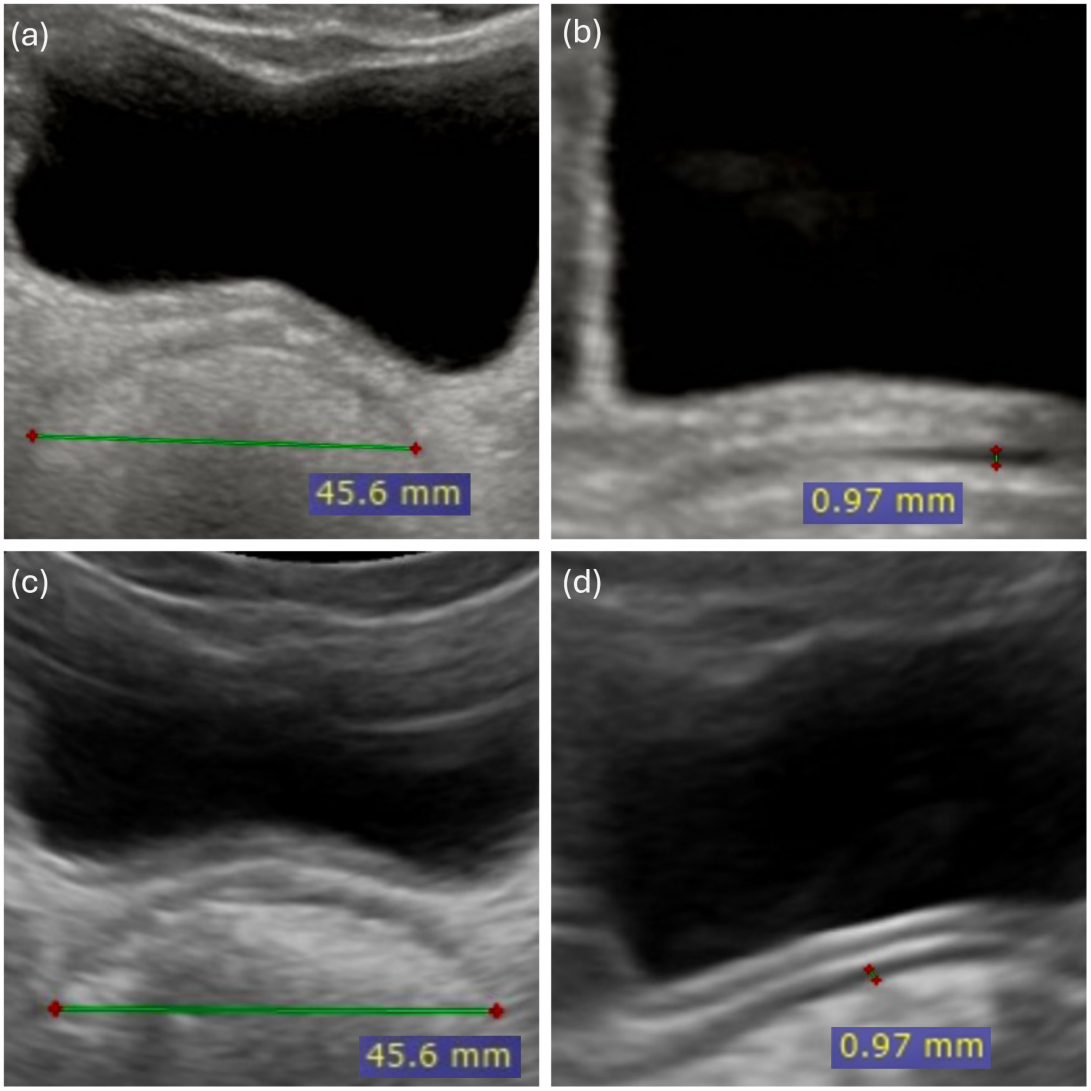
Rectal ultrasound measurements using standard and pocket-sized handheld ultra sound in a child with functional constipation. Rectal ultrasound measurements in an 8.5-year-old child with functional constipation, obtained with both standard and pocket-sized handheld ultrasound devices. Standard ultrasound: **(a)** Transverse rectal diameter (TRD) and **(b)** Rectal Anterior rectal wall thickness (RAWT). Pocket-sized handheld ultrasound (PsUS): **(c)** Transverse rectal diameter (TRD) and **(d)** Rectal anterior wall thickness (RAWT). All measurements are displayed in millimeters (mm).

The widest section of the rectal ampulla was selected from several frames for the measurement. For the TRD measurement, the cursors were placed on the outer edge of one rectal wall and the outer edge of the opposite rectal wall and the distance was measured. For the RAWT measurement, the cursors were placed at recognisable echogenic boundaries corresponding to the visible smooth muscle layer. This approach was consistently applied in all cases in this study. Figure 2 shows the TRD and RAWT measurements with a standard and a pocket-sized handheld US devices in a child with FC.

The US examinations were performed while urine was retained in the bladder to serve as an acoustic window (35). During these examinations, participants were positioned supine with their heads supported at a 10-degree angle and knees slightly flexed to reduce abdominal pressure. Images were considered suitable for analysis if they met two criteria: (1) clear bladder visualization as a well-defined anechoic region, and (2) distinct rectal tissue delineation with visible edges and margins, allowing differentiation between rectal contents, intestinal structures, and bladder wall. Data collection per session lasted approximately 10 min, with each US examination lasting approximately 2 min. Images from all four test conditions were compressed and stored for later review. The examiners were blinded to the PFC status of the patient. To evaluate the temporal stability of TRD and RAWT outcomes, 32 randomly selected participants underwent additional examination (T2) within two weeks of the initial assessment (T1) at the same site.

### 2.3 Statistical analysis

Descriptive statistics were applied to summarize participants' baseline characteristics, reported as mean and standard deviation (SD). The agreement between the novice and the expert practitioner (for both devices: conventional US/PsUS) and the validity of the PsUS compared to the conventional US were assessed using intraclass correlation coefficient (ICC) models. Specifically, a two-way random effects model with absolute agreement and multiple practitioners/measurements was used (37, 38). ICC values were interpreted as follows: <0.5 poor agreement, 0.5–0.75 moderate, 0.75–0.9 good, and >0.9 excellent (37).

To calculate the temporal stability of the TRD and RAWT measures, ICC was used to compare the results of the first (T1) and the second examination (T2), performed within two weeks (for each device and each practitioner). The standard error of measurement (SEM) and the minimal detectable change (MDC95%) were calculated as supplementary temporal stability indices. SEM was calculated as follows: SEM = SD×√(1-ICC), and MDC was determined as MDC95%=1.96×SEM×√2 (39).

To determine the optimal cut-off values for TRD and RAWT for the diagnosis of constipation, receiver operating characteristic curve (ROC) analysis and the Youden index were used with the Rome IV criteria as the gold standard. The values of TRD and RAWT used for this analysis were the average of all four measurements (both practitioners and both devices). The area under the curve (AUC) was considered appropriate when ≥0.8. Negative and positive predictive values were then calculated based on the Bayes formula with a prevalence of 12% (40). Analysis was conducted using R and RStudio software with the “tidyverse,” “irr,” and “stats” packages (41–44). A *P*-value of <0.05 was considered significant.

## 3 Results

### 3.1 Participant characteristics

The sample had a mean age of 8.3 ± 2.7 years, BMI of 16.06 ± 3.12, There were 31 males (60%) and 21 females (40%).

Constipated participants (*n* = 28) showed an increased TRD of 35.45 ± 5.31 mm compared to non-constipated participants (*n* = 24), who had a TRD of 26.64 ± 5.43 mm, [95% confidence interval (CI): 5.80–11.82, *p* < 0.001]. Similarly, the RAWT was thinner in constipated participants (1.87 ± 0.54 mm) compared to non-constipated participants (2.81 ± 0.54 mm), (95% CI: 0.63–1.24, *p* < 0.001).

### 3.2 Validity of PsUS and the inter-raters' agreement

The PsUS ICC validity results for the TRD measures were excellent (ICC > 0.9) and for RAWT good to excellent with the lowest ICC = 0.87 (95% CI: 0.78–0.92). The inter-rater agreement was excellent for all measures (TRD and RATW) under both US devices. Table 1 shows all ICC values related to PsUS validity and inter-raters' agreement.

**Table 1:** Validity of PsUS and agreement between expert and novice rater (*n* = 52).

Measurement	Outcome	Comparison	ICC (95% CI)
TRD	Validity	Expert – Conv. vs. PsUS	0.96 (0.93–0.98)
		Novice – Conv. vs. PsUS	0.96 (0.94–0.98)
	Agreement	Conv – Expert vs. Novice	0.98 (0.98–0.99)
		PsUS – Expert vs. Novice	0.99 (0.97–0.99)
RAWT	Validity	Expert – Conv. vs. PsUS	0.87 (0.78–0.92)
		Novice – Conv. vs. PsUS	0.92 (0.87–0.95)
	Agreement	Conv – Expert vs. Novice	0.98 (0.97–0.99)
		PsUS – Expert vs. Novice	0.97 (0.96–0.98)

TRD, transverse rectal diameter; RAWT, rectal anterior wall thickness; Conv, conventional ultrasound; PsUS, pocket-sized hand-held ultrasound; Expert, experienced clinician; Novice, inexperienced clinician; ICC, intraclass correlation coefficient; CI, confidence interval. All ICC values are statistically significant at *p* < 0.001.

### 3.3 Temporal stability of the TRD and RAWT measures

The TRD measurement demonstrated good stability across assessments by both novice and expert clinicians, with ICC values ranging from 0.79 to 0.85 for both US devices (see Table 2). Similarly, the RAWT thickness exhibited excellent stability, achieving ICC values greater than 0.9 (see Table 2). No practically meaningful differences in measurement stability were observed between practitioners or between the two devices.

**Table 2:** Temporal stability of rectal diameter and anterior wall thickness measurements using conventional and pocket-sized ultrasound devices, assessed by expert and novice clinicians (*n* = 32).

Measurement	Outcome T1 vs. T2	Conv		PsUS	
		ICC	SEM/MDC_95%_	ICC	SEM/MDC_95%_
TRD	Expert	0.80 (0.60–0.90)	3.41/9.47	0.80 (0.58–0.90)	3.21/8.90
	Novice	0.79 (0.58–0.90)	3.22/8.93	0.85 (0.68–0.92)	2.64/7.34
RAWT	Expert	0.93 (0.85–0.96)	0.20/0.55	0.93 (0.85–0.96)	0.19/0.55
	Novice	0.92 (0.84–0.96)	0.20/0.55	0.92 (0.84–0.96)	0.19/0.53

TRD, transverse rectal diameter; RAWT, rectal anterior wall thickness; Conv, conventional ultrasound; PsUS, pocket-sized hand-held ultrasound; Expert, experienced clinician; Novice, inexperienced clinician; ICC, intraclass correlation coefficient; CI, confidence interval; SEM, standard error of measurement; MDC95%, minimal detectable change 95% CI. All ICC values are statistically significant at *p* < 0.001.

The SEM TRD ranged from 3.21 to 3.41 mm, except for measurements taken by novice practitioners using PsUS, which showed a lower SEM of 2.64 mm. The corresponding minimal detectable change at 95% confidence (MDC_95%_) for TRD ranged from 8.93 to 9.47 mm, with a reduced MDC_95%_ of 7.34 mm for the novice practitioner using PsUS, indicating greater precision. For RAWT thickness, SEM values were consistently around 0.20 mm across both practitioners and devices. The MDC_95%_ values for RAWT also remained stable across all measurements, ranging between 0.53 and 0.55 mm.

### 3.4 Cut-off values for TRD and RAWT for the diagnosis of constipation

**Figure 3:**
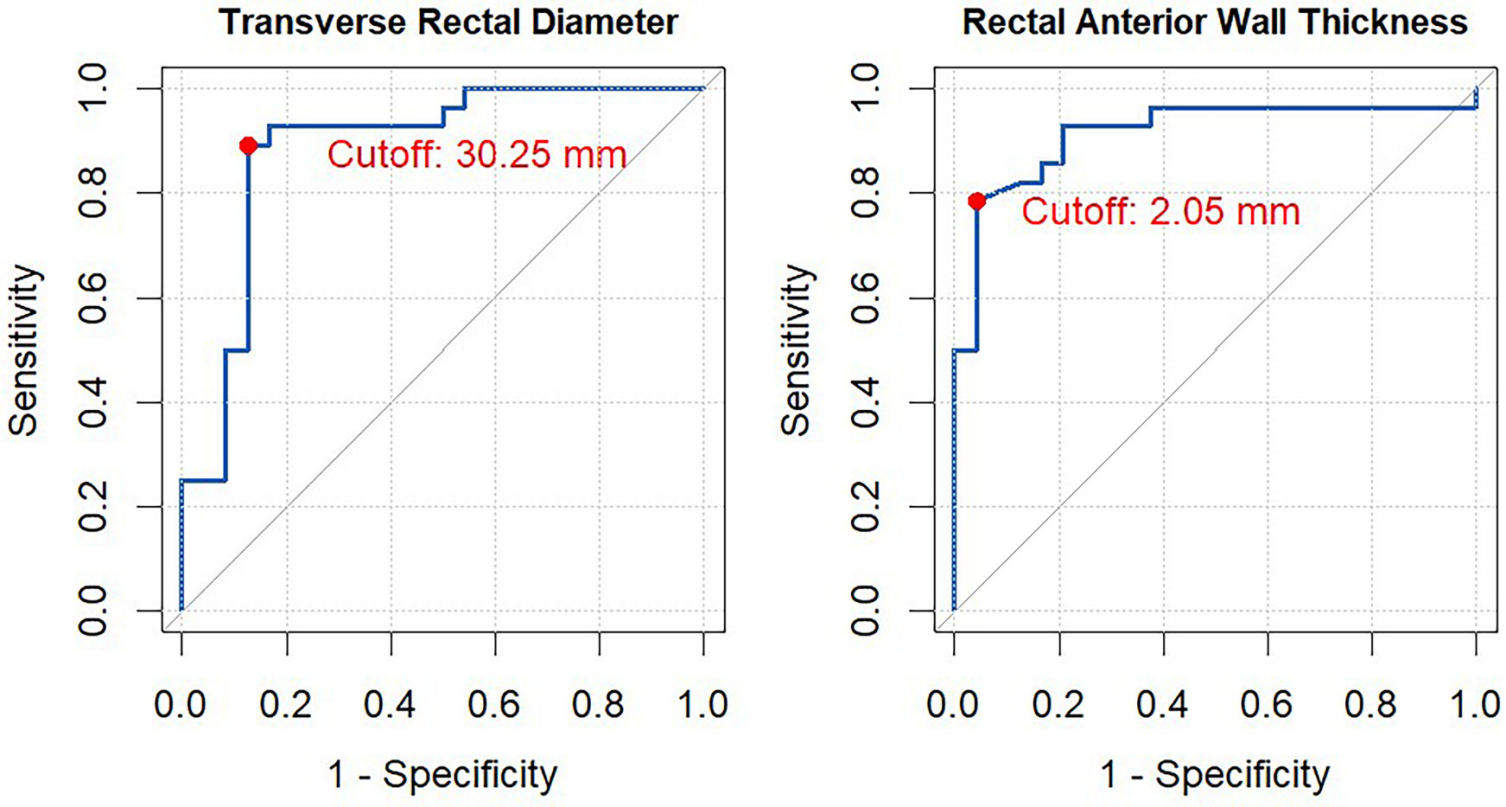
ROC curve analysis for transverse rectal diameter (TRD) and rectal anterior wall thickness (RAWT). This figure illustrates the Receiver Operating Characteristic (ROC) curve for evaluating the performance of transverse rectal diameter (TRD) and rectal anterior wall thickness (RAWT) in distinguishing between constipated and non-constipated children according to the Rome IV Criteria. The optimal cut-off point was determined using Youden's Index. In the analysis were the mean of all four measurements performed for each outcome measure.

Figure 3 presents the ROC curves and optimal cut-off values for TRD and RAWT in identifying constipation, using the Rome IV criteria as the gold standard. The AUC for TRD was 0.88 (95% CI: 0.79–0.99), with an optimal cut-off value of 30.25 mm, giving a sensitivity of 0.89 and a specificity of 0.88. The positive predictive value (PPV) for TRD was 0.49, while the negative predictive value (NPV) was 0.98.

For RAWT, the AUC was 0.91 (95% CI: 0.83–1.00), with an optimal cut-off of 2.05 mm, giving a sensitivity of 0.79 and a specificity of 0.96. The PPV and NPV for RAWT were 0.71 and 0.97, respectively.

## 4 Discussion

FC is a significant health concern that profoundly affects children's well-being. Current evaluation methods of PFC, often relying on subjective criteria and invasive procedures, have notable limitations.^44^ In this study, we explored the potential of PsUS, administered by clinicians with varying levels of experience, as a non-invasive and child-friendly approach to support the evaluation and monitoring of PFC, addressing the need for more addressing the need for more objective, accessible, and reproducible tools in pediatric gastroenterology.

Our results showed that PsUS provides valid measures of TRD and RAWT, key indicators of PFC. The validity of the PsUS for the assessment of PFC is consistent with pediatric studies from various medical settings in which PsUS and conventional US have been shown to be comparable in accuracy (37–45). Studies assessing constipation in adults have also demonstrated the validity of the PsUS (38, 39); however, to our knowledge, there are no available data on the use of PsUS in the assessment of FC in children. The present study fills this gap and provides new evidence on the use of PsUS to assess PFC.

We found strong agreement between experienced and novice clinicians in measuring TRD and RAWT. While there is no previous literature specifically addressing this comparison in the context of PFC using US techniques and PsUS in particular, similar studies in other pediatric fields, such as gastroenterology (40), cardiology (41, 42), and emergency care (43), have shown that trained novices can achieve assessment accuracy comparable to experts after appropriate training with PsUS. Our results are also consistent with those of Yabunaka et al. (39), who showed that PsUS can determine TRD for the desire to defecate in old adults with agreement between US-trained nurses and US-experienced clinicians. These results emphasize the feasibility of PsUS for broader clinical application while maintaining high measurement reliability.

Demonstrating that a physiotherapist can use PsUS after brief POCUS training may encourage other physiotherapists involved in the follow-up and management of pediatric pelvic floor conditions to pursue training and contribute to improved care for children with functional constipation.

In addition to measurement accuracy, our results also emphasize the clinical utility of PsUS. By providing objective, real-time evaluation of TRD and RAWT, PsUS facilitates continuous monitoring of rectal distension and wall thickness, allowing for individualized treatment adjustment. In addition, the non-invasive nature of this method offers a child-friendly alternative to DRE and expensive imaging procedures, minimizing discomfort and improving patient compliance. While artificial intelligence (AI) tools were not used in this study, it should be noted that emerging AI technologies are enabling less experienced clinicians in fields such as obstetrics and gynecology (45), cardiology (44) and intensive care (46) to train with PsUS. The integration of similar AI tools in pediatric gastrogeology, which provide real-time guidance for TRD and RAWT measurements and anatomic feature identification, could further improve the diagnosis and monitoring of PFC.

To confirm the suitability of a measurement for monitoring, its stability over time must be assessed. High temporal stability between measurement time points indicates that changes in the measurement are unlikely to occur without intervention, making it a reliable tool for tracking treatment progress.

Our study shows that both TRD and RAWT provide reliable and consistent results over a two-week period when measured with either PsUS or conventional US. The high ICC values observed (>0.79 for TRD, >0.92 for RAWT) confirm high reproducibility of measurements and minimal inter-observer variability, emphasizing the robustness of the PsUS in tracking rectal distension and wall thickness over time. While the temporal stability of TRD and RAWT was good over two weeks, minor differences were observed in these measurements by both clinicians, which were similar in both US devices. These slight variations in rectal US measurements can be attributed to factors such as the timing of defecation, which Modin et al. (10) found to influence TRD, and rectal distension. Song et al. (47) and Scaife et al. (48) alsoemphasized the influence of rectal volume and small changes in position during the examination. Overall, although there may be slight differences in TRD and RAWT measurements within two weeks, the strong temporal stability of these measurements supports their reliable use in clinical practice. Beyond its diagnostic value, the PsUS can play an important role in the ongoing management of PFC. Its ability to provide objective measurements in real time allows for longitudinal evaluation, facilitating timely adjustment of treatment and reducing reliance on invasive examinations, which can be impractical for repeated assessments in children. In addition, the high agreement between experts and novices underscores the consistency and reproducibility of PsUS measurements, strengthening its applicability beyond specialized sonography facilities. The results of our study on TRD and RAWT cut-offs in the diagnosis of FC in children are consistent with the existing literature, with the addition of new information. Our TRD threshold of 30.25 mm with a sensitivity of 0.89 and specificity of 0.88 is consistent with previous studies, such as those of Hamdy et al. (26) who reported TRD cut-offs of 30 mm and 25 mm for different age groups. Although the TRD cut-off of 38 mm reported by Doniger et al. (49) is slightly higher than our threshold, it falls within the MDC_95%_ for TRD (8.93–9.47 mm), indicating it is not substantially different from our findings. The RAWT cut-off value of 2.05 mm, with a sensitivity of 0.79 and a specificity of 0.96, represents a new contribution of our study and has not been previously documented. While existing research, such as the studies by Pop et al. (50) demonstrating wider RAWT in children with constipation and Shapouri et al. (24) observing increased RAWT in FC (24, 50), support the association between increased RAWT and constipation, our study is the first to propose a specific cut-off value of 2.05 mm. This finding may provide an important clinical benchmark for future assessments of RAWT in children with FC. While this RAWT cut-off requires further validation in larger studies, it represents a promising tool for assessing PFC.

Shapouri et al. (24) suggested that RAWT has less variability and a stronger association with chronic constipation. In addition, Pop et al. (50) observed a correlation between RAWT and the duration and severity of constipation. Yet, assessment based on both RAWT and TRD measures may improve diagnosis and follow up of PFC. TRD may detect acute changes, such as fecal impaction (49), while RAWT could provide information about more chronic rectal changes. Overall, our results confirm that both TRD and RAWT are suitable for the diagnosis of PFC, with the new RAWT cut-off providing additional valuable insights.

This study has several limitations that should be carefully considered. Nevertheless, it represents the first standardized evaluation of TRD and RAWT measurement technique in PFC care and shows statistically significant results with strong clinical implications. Conducting the study at a single site with a limited number of raters may limit the generalizability of the results to other clinicians, clinical settings, or populations. In addition, the high inter-rater agreement observed in our study suggests minimal inter-observer variability, further supporting the consistency and reproducibility of PsUS in assessing PFC. Our exploratory findings releated to RAWT should be interpreted with caution as the current evidence for using this outcome in pediatric populations is limited. In contrast, TRD has been studied more extensively and is considered a more established sonographic marker in the evaluation of pediatric constipation. Future studies are needed to validate these results in different clinical settings, with a larger cohort of clinicians and broader pediatric populations. In addition, the influence of factors such as age, gender and body mass index on the measurements should be further investigated. Notably, the strong correlation patterns identified in our cohort provide a solid framework for these future investigations. Finally, our comparison was limited to the conventional US examination and Rome IV criteria; the inclusion of additional diagnostic methods may allow for a more comprehensive evaluation. Future studies should address these aspects and further investigate whether the integration of PsUS into routine pediatric practice can improve outcomes and reduce the need for invasive assessments.

## 5 Conclusions

This study demonstrates that PsUS is a valid and reliable tool for evaluating and monitoring PFC. We demonstrated that PsUS is as accurate as conventional US in measuring TRD and RAWT with high agreement between experienced and novice clinicians. Additionally, our findings provide reference values to aid in the assessment of PFC. The portability, affordability, and ease of use of PsUS, requiring minimal training, have the potential to expand access to accurate diagnosis and follow-up in PFC, ultimately enhancing patient care and outcomes for this prevalent condition.

## Data Availability

The raw data supporting the conclusions of this article will be made available by the authors, without undue reservation.
